# A direct observation method for auditing large urban centers using stratified sampling, mobile GIS technology and virtual environments

**DOI:** 10.1186/s12942-017-0079-7

**Published:** 2017-02-16

**Authors:** Sean J. V. Lafontaine, M. Sawada, Elizabeth Kristjansson

**Affiliations:** 1 School of Psychology, University of Ottawa, Ottawa, ON K2L 1K9 Canada; 2 Laboratory for Applied Geomatics and GIS Science (LAGGISS), Department of Geography, Environment and Geomatics, University of Ottawa, Ottawa, ON K1N 6N5 Canada; 3 Ottawa Neighbourhood Study (ONS), University of Ottawa, Vanier 5023, 136 Jean Jacques Lussier, Ottawa, ON K1N 6N5 Canada; 4 School of Psychology and Institute of Population Health, University of Ottawa, Ottawa, ON K2L 1K9 Canada

**Keywords:** Direct observation, Auditing, Large urban centers, Methodological approach, Stratified sampling, Technology, Virtual environments

## Abstract

**Background:**

With the expansion and growth of research on neighbourhood characteristics, there is an increased need for direct observational field audits. Herein, we introduce a novel direct observational audit method and systematic social observation instrument (SSOI) for efficiently assessing neighbourhood aesthetics over large urban areas.

**Methods:**

Our audit method uses spatial random sampling stratified by residential zoning and incorporates both mobile geographic information systems technology and virtual environments. The reliability of our method was tested in two ways: first, in 15 Ottawa neighbourhoods, we compared results at audited locations over two subsequent years, and second; we audited every residential block (167 blocks) in one neighbourhood and compared the distribution of SSOI aesthetics index scores with results from the randomly audited locations. Finally, we present interrater reliability and consistency results on all observed items.

**Results:**

The observed neighbourhood average aesthetics index score estimated from four or five stratified random audit locations is sufficient to characterize the average neighbourhood aesthetics. The SSOI was internally consistent and demonstrated good to excellent interrater reliability. At the neighbourhood level, aesthetics is positively related to SES and physical activity and negatively correlated with BMI.

**Conclusion:**

The proposed approach to direct neighbourhood auditing performs sufficiently and has the advantage of financial and temporal efficiency when auditing a large city.

## Background

The impact of qualitative characteristics of the built environment (BE) on health and well-being has become well established in health geography [1–3]. At the neighbourhood level, the impact of the built environment on physical and mental health has provided evidence of the link between urban disorder and social status [4, 5]. Establishing such linkages forms the basis for evidence based decision making that can improve neighbourhoods and the well-being of residents. Given the potential importance of such evidence for neighbourhood renewal efforts and policy formulation, there is a fundamental need to evaluate the methods, context, and manner through which such research is completed. There have been recent efforts to evaluate new methods of qualitative observations of neighbourhood BEs using new approaches and technologies [6–12]. This study adds to those efforts by presenting an efficient and effective method of undertaking qualitative neighbourhood observations over large urban areas using mobile GIS technology.

Neighbourhood level BE audits use a wide range of data collection techniques to gather information about the contextual factors that can affect residents. Common data collection techniques include observations of resident perceptions (via phone interviews or mailed questionnaires) or secondary use of census data. However, results based on resident perceptions can contain response bias and census data are limited to information on neighbourhood socio-economic structure and rarely capture information on the BE qualitative characteristics [13–15]. The most effective approach to BE auditing is direct observational research. Direct observation of the BE allows for the collection of fine-grained details at various spatial scales. However, few studies have used direct observational data collection techniques to evaluate neighbourhood characteristics over entire urban centers [5]. Auditing large urban centers is a daunting task; direct observational field audits require auditors (two or more for reliability assessment) to be physically present to evaluate and observe the built environment at multiple locations. Large-scale spatial audits can be time and cost intensive [5, 16, 17]. For example, in one of the largest direct observation studies in Canada, researchers physically audited a total of 176 block faces across six Toronto neighbourhoods over 3 months (August–October) [5]. Even such a relatively modest sized direct observational study presents considerable financial and temporal constraints. Thus, extending a direct audit to an entire large urban center, block-by-block, is beyond the financial capacity of modestly funded research projects. Time and financial expediency underline the need to develop more efficient methods for direct field audit studies.

In response to such practical limitations on direct observation, and with varying degrees of success, some studies have employed vehicles or vehicle-mounted video recordings to achieve rapid auditing of the BE [18–20]. The use of a virtual environment (VE) such as Google Street View or Microsoft StreetSide is increasingly being explored in lieu of real-time built environment audits [1, 11, 15–17, 19, 21–26]. For example, a systematic social observation instrument (SSOI) applied using both Google Street View and a direct field audit for 143 items across 37 block faces in New York City, found strong concordance for some dimensions of walkability, but only modest agreement for aesthetics and physical disorder [23]. In other cases, strong correlations between virtual and field audits for items such as recreation, the food environment and land use have been observed [1]. VE audits using Google Street View, panoramic imagery or video footage do show high interrater reliability [21, 26]. Even crowd sourcing is being explored as a means to distinguish between perceived safety, class and uniqueness of city blocks [27]. Some research has employed machine based learning to assess perceived qualities of the BE such as safety and walkability [28].

Although BE virtual audits have met with some success, they cannot match the depth and comprehensiveness of direct real-time observations [11]. Why? Because VEs do not feed a number of sensory inputs [16, 29] including noise levels, soundscape and scent among others. Moreover, within a VE like Google Street View, the date of image acquisition can change suddenly and unpredictably, particularly across intersections [22] and cause temporal discrepancies (year or season) that bias audit results—either human or machine based. Virtual audits are also limited in measuring fine-grained or micro-level detail in images [1, 15–17, 23]. A balance between direct and virtual BE audits may be achieved by mixed methods that utilize technology to achieve temporal and financial efficiency, while maintaining the integrity and comprehensiveness of real-time observation methods.

To what degree can a combination of mobile GIS technology and limited spatial sampling adequately assess, with minimal time and effort, qualitative neighbourhood characteristics across large urban areas? To address this question, this study presents and evaluates a novel direct observation method that employs a simple SSOI to assesses urban aesthetics. However, the focus of this research is not on the instrument itself. Rather, this study focuses on the performance of a BE audit method that combines VEs for auditor training and mobile GIS technology for real-time data collection at randomly audited locations within neighbourhoods. We assess whether our audit method is sufficient to measure the qualitative variability of the BE across neighbourhoods in a large urban center. The accuracy of the random sampling design is assessed by comparing results to a complete block-by-block audit of 167 block faces in one of the neighbourhoods. Internal consistently and interrater reliability are calculated for all raters. The proposed method holds considerable promise as a means to conduct spatial large-scale audits of the BE that can add important independent variables for health geographic studies.

## Methods

### Audit instrument

Our goal was not to produce an exhaustive systematic social observation instrument (SSOI), rather, we simply wanted to produce an SSOI scale that would be sufficient in measuring the variation in aesthetic quality across the BE. The items were selected and the scale was developed after reviewing literature that used measurement scales aimed to assess components of the environment. To increase the breadth and depth of measures and approaches to measurement scale development, we included studies that were not solely focused on aesthetics. Relevant studies [5, 18, 30–36] were examined and organized by reviewing content, domains, measures, items, data collection, and psychometric properties. We also included research conducted in North America and Europe in our review of the literature that provided a diversity of geographic locations [4, 5, 30, 31]. Furthermore, we used an approach employed by Caughy et al. [18] and Parsons et al. [5] among others, in which pilot testing was used to further refine the SSOI items. This process lead to the development of a 10 item scale (Table 1) with each item having five Likert response values.

**Table 1:** Each SSOI item contained five Likert response values: extremely poor, below average, average, above average, and excellent (for qualitative items) or none, few, some, many and lots (for quantitative items)

Item	Auditor 1	Auditor 2	Mean
Cleanliness of streets and properties	0.100	0.152	0.126
Presence of trees	0.038	0.091	0.064
Quality of trees	0.049	0.050	0.049
Landscaping	0.220	0.086	0.153
Flowers and shrubs	0.079	0.047	0.063
Houses well-spaced	0.085	0.204	0.144
Upkeep of homes	0.284	0.244	0.264
Presence of outdoor furniture	0.037	0.032	0.034
Quality of outdoor furniture	0.051	0.034	0.042
Pedestrian infrastructure	0.058	0.061	0.059

Item weightings used in deriving the aesthetics index score, $s_{i \cdot }$, for each audit location were determined as the mean value from both auditors—see text for details

The creation of descriptors for the Likert response values for each item was a vital step in the development of an SSOI for many reasons, but most importantly because of the subjective nature of observations [1, 5]. Each item’s Likert response scale contained three descriptor definitions (a descriptor for the maximum value, middle value and lowest value) together with reference photos for each value. The instrument itself was entered on a mobile GIS device so that data would be collected and validated in real-time.

### Mobile GIS technology

**Fig. 1:**
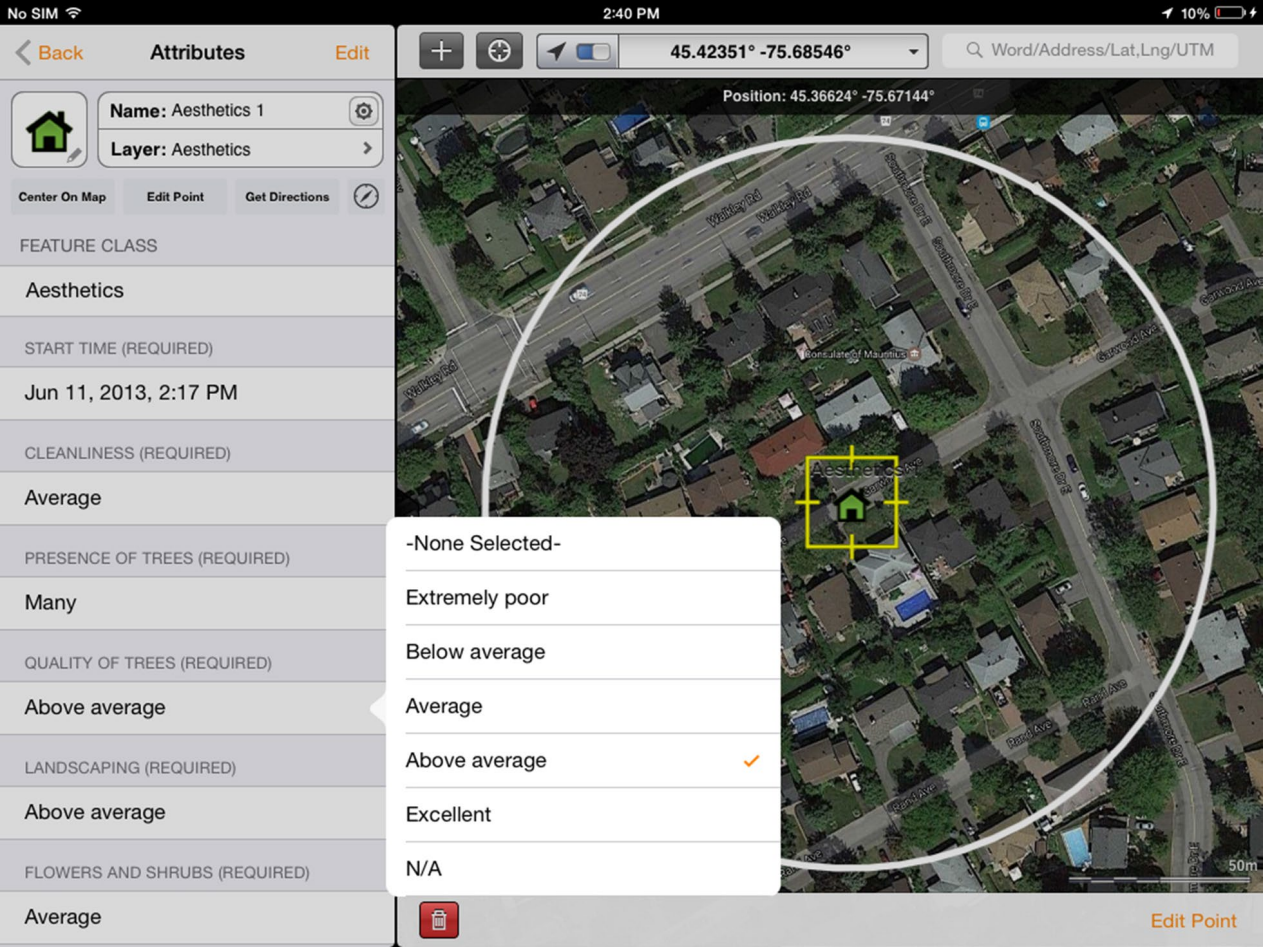
Main data entry screen of GIS Kit Pro on iPad. *Left pane* provides SSOI items and pick-lists for Likert response values. *Right pane* provides current GPS position and offline Google satellite view as well as the audit location as a point feature at which the Likert response values will be stored. Observation buffer zone is shown and was loaded from a shapefile created in a desktop GIS

Mobile devices with GPS receivers provide a platform for rapid and comprehensive data collection [37]. An Apple iPad 2 + cellular was chosen for this research because the ‘+cellular’ models contain the hardware-based GPS receiver required to record positions of field audit points without an internet connection (off-line mode). We used GIS Kit Pro by GARAFA software for mobile mapping and data collection on Apple’s iOS (Fig. 1). The GIS Kit application is a mobile Geographic Information System (GIS) software that combines data management with a mapping engine for an effective mobile data solution (see http://www.garafa.com). The single-use license fee for GIS Kit was $299 per user and an iPad + cellular was ~$599. The aesthetics SSOI was entered into GIS Kit as a feature class.

The process of collecting, transferring and processing data using a mobile device takes 50% less time when compared to traditional paper-based methods [24, 38]. While time is not reduced when undertaking observations, the expediency originates from the reduced data processing and handling provided by an all-digital approach. As such, field audit data requires no post-field transcription or geo-referencing. In comparison to a complete VE audit, the only appreciable difference is the time taken to travel between audit locations with the mobile GIS technology. In this research, the data collected within GIS Kit were exported as shapefiles and directly opened in a desktop GIS, Google Earth or within a statistical analysis package.

### Sampling strategy

**Fig. 2:**
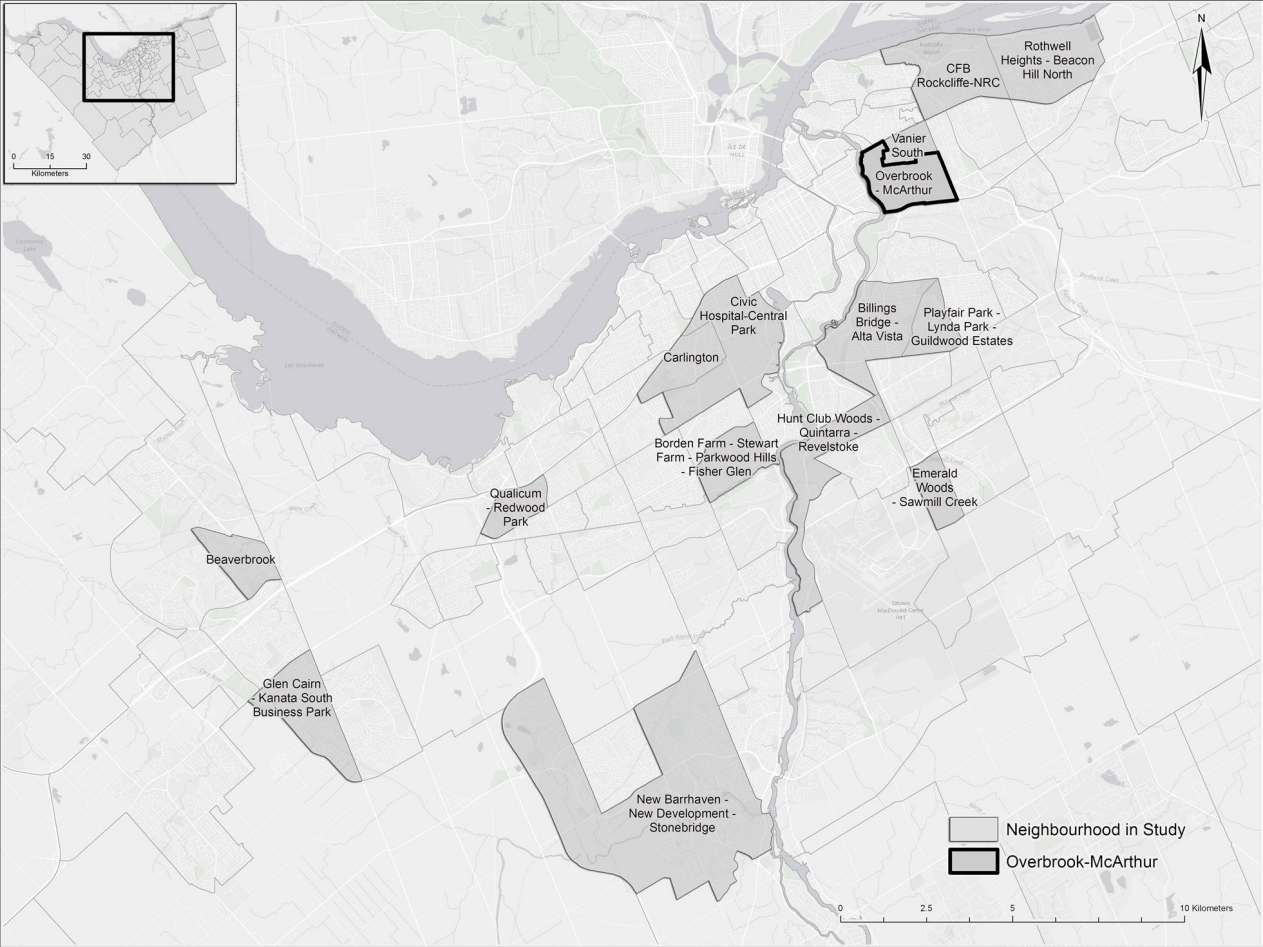
Fifteen neighbourhoods included in study. The Overbrook-McArthur neighbourhood was observed at each block face as described in the text and in Fig. 4

**Fig. 3:**
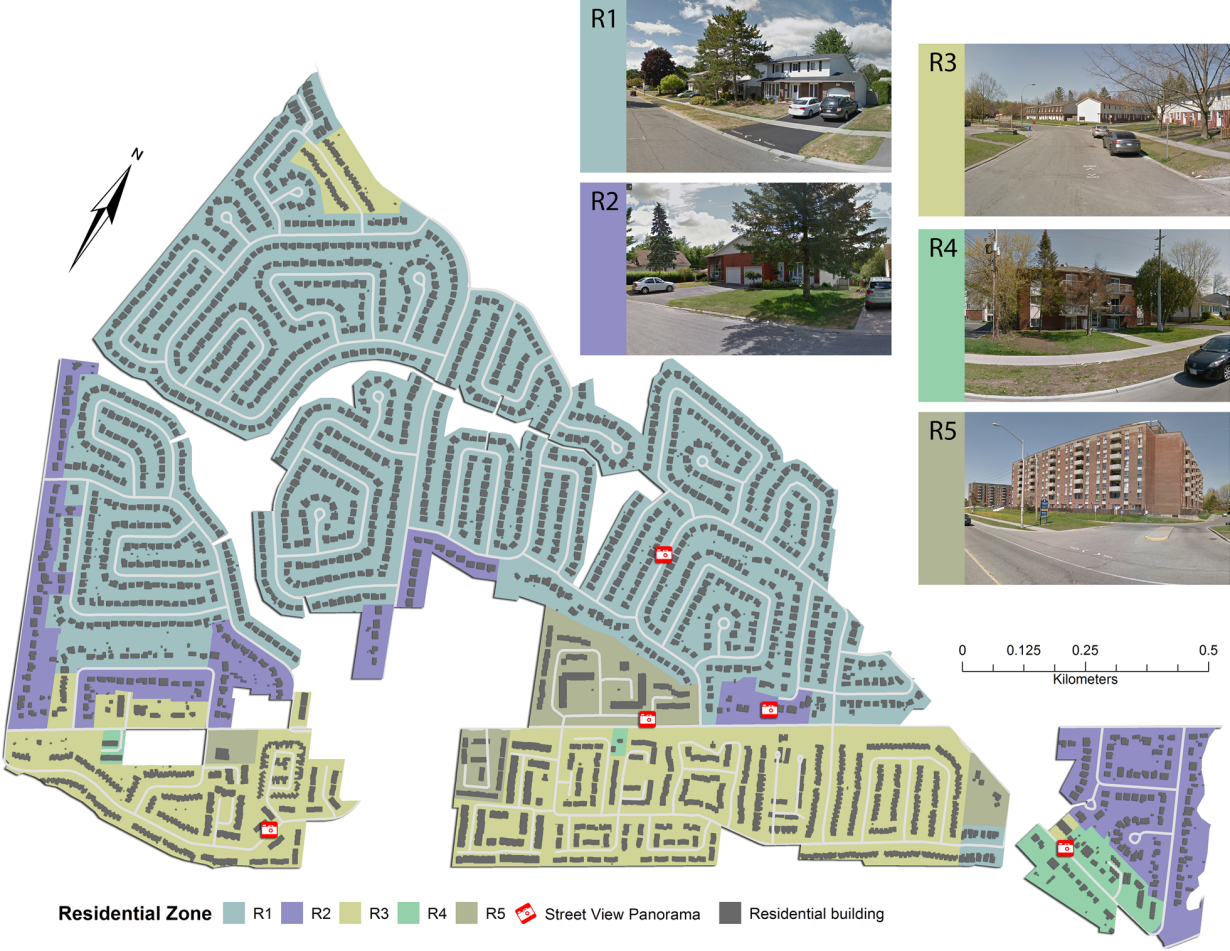
Schematic map of five residential zoning types used to determine audit locations illustrated with an example neighborhood within the study region. Building footprints illustrate relative residential housing density. The example Street View Panoramas (© 2016 Google) from these zones illustrate typical property types. Zone RM (mobile home) is not included because of its rarity over the entire study area

Field audits took place within 15 neighbourhoods (Fig. 2) selected from the Ottawa Neighbourhood Study (ONS) (www.neighbourhoodstudy.ca). We based this selection on neighbourhood SES quintile; we selected 5 high, medium, and low neighbourhoods. Audit points within each of these neighbourhoods were located based on residential zones defined by City of Ottawa by-laws: R1—Residential First Density (detached dwellings), R2—Residential Second Density (two unit dwellings), R3—Residential Third Density (multiple attached dwellings), R4—Residential Fourth Density (low rise apartments), R5—Residential Fifth Density (mid/high-rise apartments) and the RM-Mobile Home (Retrieved from http://www.ottawa.ca/residents/bylaw/a_z/zoning/parts/pt_06/index_en.html) (Fig. 3). Within Ottawa, high density zoning (tower blocks and multiunit apartments, R4 and R5 in Fig. 3) can be indicative of lower income areas when compared to low density residential zoning (single family homes to town homes, R1–R3 in Fig. 3). In the absence of highly resolved socioeconomic data at the sub-neighbourhood block-level that could be used to guide the determination of audit locations within neighbourhoods, the probability of selecting an audit point was made directly proportional to the area occupied by each residential zone type within a neighbourhood. Here, we are loosely assuming that residential zoning density is a proxy variable for within-neighbourhood variation in SES. Within each of the 15 neighbourhoods, four (2011) or five (2012) audit points were located. Overall, there were 60 (2011) and 90 (2012) audit points across the 15 Ottawa neighbourhoods. At each audit point, a 100 m buffer (radius of circle) was created within a desktop GIS and the buffers were loaded into the mobile GIS Kit. These buffers represent the audit locations within which observations are made. The GPS capability of the iPAD + Cellular allowed the auditor to actively monitor their position on the mobile map within GIS Kit and thereby determine when they arrived at the edge of an audit location to begin observations (Fig. 1). To control for the variability in block length and observation time across the urban area, observations were only made within the audit location (e.g., within each audit point’s buffer zone).

**Fig. 4:**
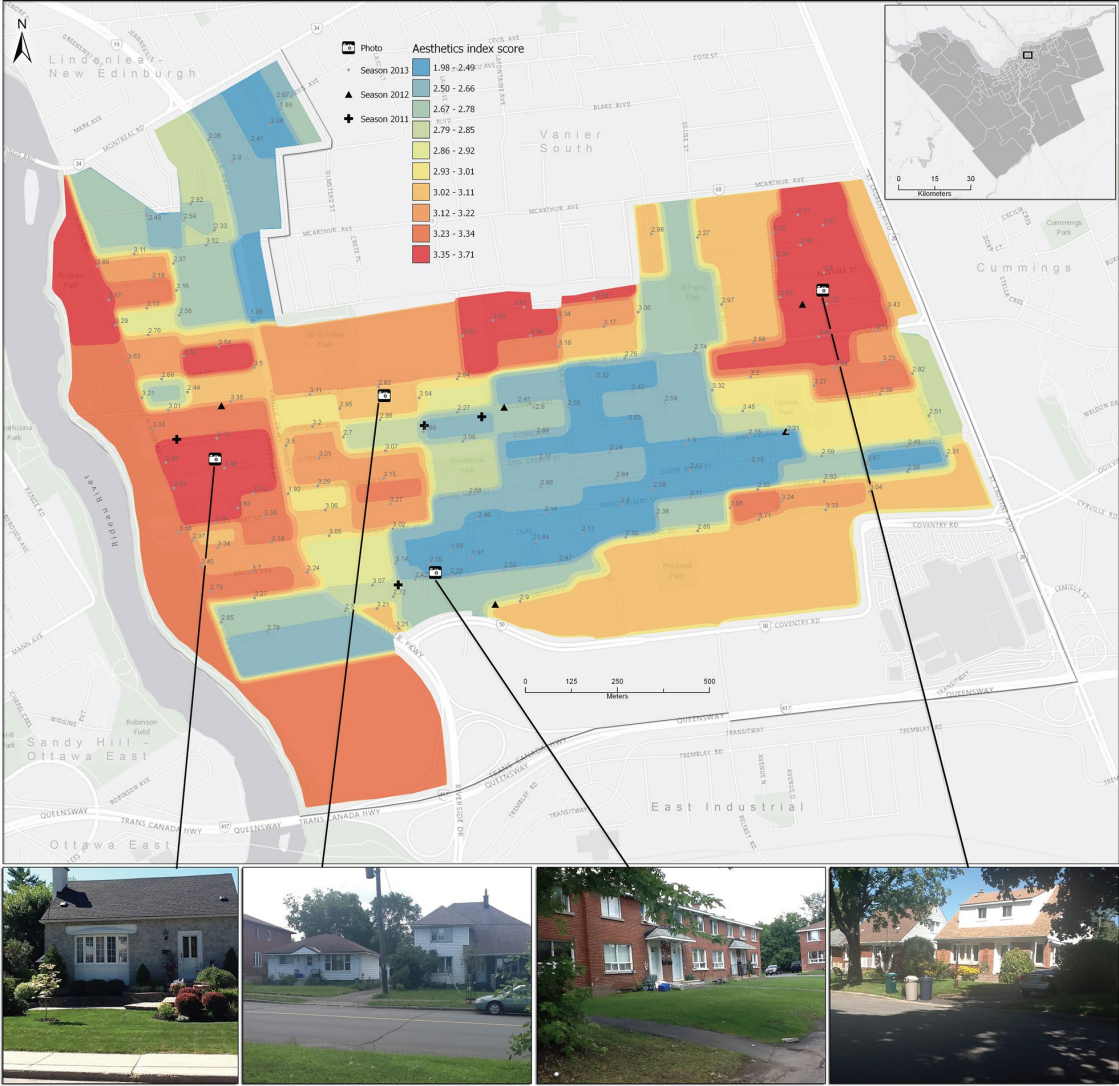
Overbrook-McArthur neighbourhood with pycnophylactic surface (see text) of average block aesthetic index scores $\left( \bar{{s_{i \cdot } }} \right)$. The audit locations are shown as points, with the calculated $s_{i \cdot }$ (in *light gray* font, *top right* of each audit location). Audit locations in 2011 (*n* = 4) and 2012 (*n* = 5) are shown for reference. Google Street View (© 2015 Google) panoramas represent example block faces in areas with different $s_{i \cdot }$ values. Basemap: Esri, HERE, DeLorme, MapmyIndia, © OpenStreetMap contributors, and the GIS user community

To validate the sensitivity of results to the number of audited locations in a neighbourhood, in 2013 every residential block (167 blocks) within one neighbourhood was audited (Fig. 4). We then compared the average and frequency distribution of aesthetics index scores (derivation explained below) from the 167 audit locations in 2013 to the neighbourhood average aesthetics index score from the 2011 and 2012 audit locations in that neighbourhood. Additionally, for visualization purposes, the 167 block observations were used to calculate 102 average block aesthetics index scores. Pycnophylactic interpolation [39, 40], a volume preserving technique, was used to create a surface of average block aesthetics index score variation within the neighbourhood. Because our needs were only visual, a simple pycnophylactic areal interpolation technique honors the discontinuous nature of observations that apply to an entire block, while at the same time, smoothing the hard discontinuities between blocks that share block-face aesthetics index scores. However, a number of techniques for interpolation of areal data have been developed [41–44]. Many of these techniques provide estimates of uncertainty and would be more appropriate when the purpose of mapping is area-based estimation at unknown locations of a social surface or for transferring data from one zonal geography to another.

Finally, for the 15 neighbourhoods, we compared the neighbourhood average aesthetics index score results to select health determinants in order to provide impetus for research situating urban aesthetics within an ecological framework for geographic health determinants.

### Audit timing

In 2011, audits were completed between November and December on Saturday’s between 9:00 am and 5:00 pm. Each week, three or four neighbourhoods were observed. In 2012, audits were collected for 79 neighbourhoods (a subset of 15 are included here for comparison with 2011) between June and August each weekday, ensuring that data collection did not take place the day prior to, or of, garbage pick-up. Complete VE audits of the BE cannot control for the timing of garbage collection. In 2013, audits took place daily from mid-July to mid-August for each of 167 blocks in one neighbourhood (Overbrook-McArthur).

### Observational method

At each audit location, two independent observers were used in each year. Overall, six different observers were used over the three year period, two independent observers in each year. Once at the end an audit location, the two auditors would cross to the other side of the street and continue walking back to the original starting point. Immediately after walking, both auditors, independently and without discussion or debrief, completed the SSOI separately on their individual iPads. Once complete, GIS Kit saved the completed audit as a point feature together with the associated SSOI attribute data. The data was exported daily to a Dropbox account or emailed directly from GIS Kit as both *kml* and shapefiles. This method of data collection was practiced and applied consistently to all neighbourhood observations each year.

Additionally, while walking the audit location, the auditors would document certain aspects by taking geotagged photographs that were stored as attributes within the feature table for each audit point within GIS Kit. For instance, if there was an exceptional or poorly maintained property or an attractive arrangement of flowers and shrubs, one of the auditors would take a picture. This allowed the auditors to collect tangible photographic evidence of neighbourhood characteristics in addition to the Likert response values for each audit location. All photographs were collected for research purposes only; no images are made public. Additionally, no identifying features, such as addresses, were captured.

### Virtual environment training

In all years, Google Street View was used to train the auditors. Practice observation sessions (excluding the neighbourhoods in the study sample) were completed using the same SSOI and tools (iPad with GIS Kit) used for real-time field observations. A two week period of training was utilized. During the first week of training, both auditors examined the SSOI item definitions and reference images for each item’s Likert response value. Then, the raters used Google Street View to undertake practice audits at predetermined locations. Virtual training was followed by calculation of inter-rater reliability measures and an intervention session for free discussion of items that showed poor agreement. Following approximately three to four rounds of virtual practice, the auditors undertook field trials in a neighbourhood close to the University of Ottawa (Sandy Hill) before beginning real-time field data collection.

### Aesthetics index scores

Aesthetics index scores for each neighbourhood were derived as weighted averages to address the issue of the relative or subjective significance of certain items compared to others (e.g., upkeep of homes versus the presence of outdoor furniture). Weighted averages provide an additional step to reduce potential biases or preferences of different auditors. Weights were derived through the use of a pairwise comparison matrix for each of the SSOI items [45]. This comparison matrix was completed by each of the auditors after discussion and consideration. For instance, the upkeep of homes is given more weight because it has a greater impact on the aesthetics of a neighbourhood than does the presence of outdoor furniture (Table 1).

Given a rectangular matrix of a neighbourhood’s Likert response values across all SSOI items, *x*, and an equally sized matrix of row-standardized weights, *w*, a *neighbourhood average aesthetics index score*, $Q_{s}$, is calculated as:

$$\begin{aligned} s_{i \cdot } & = \frac{C}{{N_{i} }}\sum\limits_{j = 1}^{{N_{i} }} {w_{ij} x_{ij} \left\{ {\begin{array}{*{20}c} {0 \le w_{i} \le 1;\sum\nolimits_{i = 1}^{n} {w_{i} = 1} } \\ {x \in \{ 1,2, \ldots ,5\} } \\ \end{array} } \right.} \\ Q_{s} & = \frac{1}{n}\sum\limits_{i = 1}^{n} {s_{i \cdot } \left\{ {i \in \{ 1,2, \ldots ,15\} } \right\}} \\ \end{aligned}$$

where $s_{i \cdot }$ is the *aesthetics index score* for audit location *i* in neighbourhood *s*, *C* is the number of SSOI items, *C* = 10 in this study, $N_{i}$ is number of numbers in the ith row (e.g., the number of applicable items for an audit location), $w_{ij}$ is the weight for SSOI item *i* column *j*, $x_{ij}$ is the ordinal value of SSOI Likert response item *i,* column *j*. This aesthetics index score, $s_{i \cdot }$, calculation includes only $N_{i}$ so that the neighbourhood average aesthetics index score, $Q_{s}$, is not penalized for non-applicable SSOI items at a given audit location. The aesthetics index score, $s_{i \cdot }$, is normalized to range between 1 and 5 by the constant *C*.

### Internal consistency

Using the weighted matrix of auditor’s observations, we examined the internal consistency of the data to ensure validity. Internal consistency is defined as the degree of reliability within a test; the extent to which different items are assessing the same construct [46]. For the aesthetics SSOI, internal consistency was measured using Cronbach’s α with bootstrapped confidence intervals calculated using the R library ‘psych’ [47].

### Interrater reliability

Interrater reliability (IRR) measures the degree of similarity/agreement of observations made by different auditors on the same set of objects after controlling for disagreement due to observational error [48]. IRR was assessed using a two-way mixed, absolute, average-measures intraclass correlation coefficient (ICC), *r* [49]. IRR was calculated using the R library ‘irr’ [50].

### Comparison with health determinants

We compare the neighbourhood average aesthetics index score, $Q_{s}$, to three health determinants: Neighbourhood SES, self-reported overweight or obese BMI and physical activity. Neighbourhood SES was calculated for 96 Ottawa Neighbourhoods using five age-sex standardized variables from the 2006 Canadian long-form Census [51]: percent of households below the low-income cut-off, average household income, percent of unemployed residents, percent of residents with less than a high school education, and percent of single-parent families. The SES index was *t*-scored to represent a mean of 50 with a standard deviation of 10 and values for the 15 neighbourhoods were ranked from highest (1) to (15) lowest for comparison with the ranked $Q_{s}$. Physical activity was evaluated with data from previous research (unpublished) using International Physical Activity Questionnaire (IPAQ) and included self-reported overweight or obese BMI and physical activity (moderately or highly active) [52]. Relations between $Q_{s}$ in 2011 and 2012 and health determinants were established using Spearman’s rank correlation coefficient, ρ, using the R library ‘Hmisc’ [53]. Given the small sample size of *n* = 15 neighbourhoods, empirical *p*-values were calculated to assess the significance of correlation coefficients with health determinants using 9999 permutations of the independent variable ($Q_{s} \;in\; 2011, 2012)$. Bias corrected (BCa) rank correlation confidence intervals at the 95% level were determined using nonparametric ordinary bootstrapping with 10,000 iterations within the R library ‘boot’ [54, 55]. All other confidence intervals for variables presented in this paper are based on 2000 bootstrapped iterations.

All statistical analyses were undertaken in R v3.2 [56] and SPSS 22 [57] (for SES).

## Results

### Internal consistency

The reliability of all SSOI items was, in 2011, α = 0.73 (95% CI [0.63, 0.79]) and, in 2012, α = 0.64 (95% CI [0.57, 0.69]). In 2013, the full block-by-block audit in one neighbourhood yielded an α = 0.72 (95% CI [0.69, 0.75]). One SSOI item, pedestrian infrastructure, showed a very low item-total correlation in both years for the 15 neighbourhood audit (Table 2). In 2012, cleanliness, the presence of trees and quality of trees have lower item-total correlations than in the other two years (Table 2).

**Table 2:** SSOI items and comparison of internal consistency: α is Cronbach’s alpha if an item is dropped; ITC is the item-total correlation corrected for item overlap and scale reliability

Item	2011		2012		2013	
	α	ITC	α	ITC	α	ITC
Cleanliness of streets and properties	0.69	0.47	0.60	0.45	0.64	0.71
Presence of trees	0.70	0.61	0.64	0.27	0.72	0.28
Quality of trees	0.71	0.71	0.64	0.31	0.72	0.34
Landscaping	0.66	0.79	0.53	0.82	0.61	0.86
Flowers and shrubs	0.70	0.69	0.58	0.87	0.67	0.83
Houses well-spaced	0.72	0.43	0.64	0.35	0.74	0.24
Upkeep of homes	0.69	0.66	0.60	0.61	0.65	0.73
Presence of outdoor furniture	0.73	0.40	0.65	0.09	0.73	0.19
Quality of outdoor furniture	0.73	0.50	0.63	0.47	0.70	0.66
Pedestrian infrastructure	0.75	0.12	0.64	0.19	0.74	0.05

### Interrater reliability

Considering only statistically significant values, average Intraclass Correlation Coefficients (ICC) across all SSOI items in 2011 was *r* = 0.85, in 2012, *r* = 0.72 and in 2013, *r* = 0.71. In 2012, cleanliness, presence of trees and quality of trees were not significant and pedestrian infrastructure was not significant in 2013. Quality of trees in 2013 is significant with a fair IRR. The ICC values are good to excellent for all other SSOI items in all years (Table 3) [48].

**Table 3:** Interrater reliability results as intraclass correlation coefficients, *r*, for all three field seasons for 10 SSOI items retained after 2011

Item	2011				2012			2013		
	n	*r*	95% CI		n	*r*	95% CI	n	*r*	95% CI
Cleanliness of streets and properties	56	*0*.66**	[0.44,0.89]		73	0.30^#^	[−0.08,0.55]	167	0.81	[0.75,0.86]
Presence of trees	59	0.89	[0.78,0.94]		74	0.48^#^	[0.30,0.62]	167	0.75	[0.66,0.82]
Quality of trees	59	0.77	[0.59,0.87]		74	0.09^#^	[−0.31,0.32]	167	0.40**	[0.21,0.54]
Landscaping	57	0.79	[0.61,0.90]		74	0.69	[0.54,0.79]	167	0.79	[0.74,0.84]
Flowers and shrubs	57	0.78	[0.59,0.89]		74	0.66	[0.46,0.78]	165	0.80	[0.75,0.85]
Houses well-spaced	59	0.89	[0.76,0.95]		74	0.77**	[0.62,0.89]	158	0.73	[0.60,0.82]
Upkeep of homes	58	0.88	[0.80,0.93]		74	0.77	[0.64,0.84]	160	0.78	[0.71,0.84]
Presence of outdoor furniture	51	0.97	[0.93,0.99]		72	0.67	[0.48,0.79]	167	0.62	[0.47,0.72]
Quality of outdoor furniture	59	0.96	[0.88,0.99]		54	0.67	[0.47,0.81]	123	0.67	[0.57,0.76]
Pedestrian infrastructure	58	0.87	[0.76,0.94]		73	0.80	[0.66,0.88]	165	0.34^#^	[0.26,0.42]

^#^Not significant at p < 0.05, ** significant at p < 0.01, all others significant at p < 0.001

### Neighbourhood average aesthetics index scores ($$Q_{s} )$$ in 2011 and 2012

The relative ranking of $Q_{s}$ varied between 2011 and 2012 (Table 4). However, across both years, five of the neighbourhoods are consistently ranked in the lower half of the $Q_{s}$ ranks (Table 4). Alternatively, three were consistently ranked in the top five (Table 4).

**Table 4:** Neighbourhoods ranked according to neighbourhood average aesthetic index scores ($\varvec{Q}_{\varvec{s}} )$ (values are rounded to two decimal places)

Field season 2011	$Q_{s}$	Field season 2012	$Q_{s}$
Carlington	2.92	CFB Rockcliffe−NRC	2.83
Vanier South	3.02	Vanier South	2.90
Overbrook—McArthur	3.10	Glen Cairn—Kanata South Business Park	2.91
Emerald Woods—Sawmill Creek	3.28	Carlington	3.02
Civic Hospital—Central Park	3.50	Emerald Woods—Sawmill Creek	3.07
Qualicum—Redwood Park	3.61	Overbrook—McArthur	3.09
Borden Farm—Stewart Farm—Parkwood Hills—Fisher Glen	3.63	Civic Hospital—Central Park	3.19
Glen Cairn—Kanata South Business Park	3.71	Beaverbrook	3.22
Playfair Park—Lynda Park—Guildwood Estates	3.73	Hunt Club Woods—Quintarra—Revelstoke	3.29
CFB Rockcliffe-NRC	3.81	Qualicum—Redwood Park	3.31
Rothwell Heights—Beacon Hill North	3.85	New Barrhaven—New Development—Stonebridge	3.37
Hunt Club Woods—Quintarra—Revelstoke	3.89	Borden Farm—Stewart Farm—Parkwood Hills—Fisher Glen	3.38
Billings Bridge—Alta Vista	4.05	Rothwell Heights—Beacon Hill North	3.54
Beaverbrook	4.23	Billings Bridge—Alta Vista	3.56
New Barrhaven—Stonebridge	4.29	Playfair Park—Lynda Park—Guildwood Estates	3.74

Discrepancies between the 2011 and 2012 $Q_{s}$ rankings included CFB—Rockliffe-NRC which had the lowest rank in 2012 but a much higher rank in 2011. Beaverbrook was also discrepant due to the lower landscaping Likert response values in 2012 compared to 2011. A similar explanation was apparent for Glen Cairn—Kanata South Business Park. However, the rank correlation between $Q_{s}$ in both years is positive and significant 0.51 (p = 0.0241), 95% CI [0.0273, 0.8226].

### Validation of sampling approach

The full neighbourhood audit in 2013 (of Overbrook-McArthur) shows the variability and spatial structure of the aesthetics index scores, $s_{i \cdot }$, among the 167 block observations (Fig. 4).

**Fig. 5:**
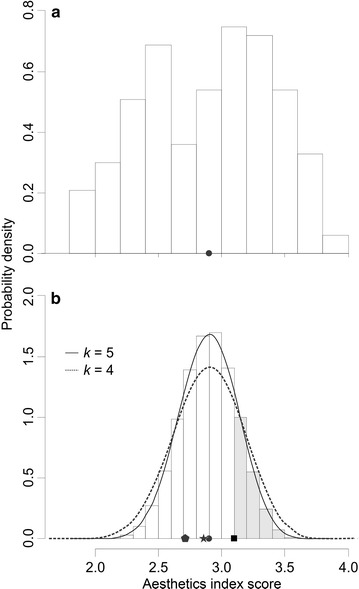
**a** Distribution of 167 aesthetic index scores ($s_{i \cdot }$) for Overbrook—McArthur neighbourhood in 2013; **b** Distribution of $Q_{s}$ for *k* = 5 randomly selected $s_{i \cdot }$ from **a**, with density estimate (*solid line*) and density estimate from distribution of *k* = 4 randomly selected $s_{i \cdot }$ from **a** (*dotted line*). *Solid black circle* is $Q_{s}$ of all 167 $s_{i \cdot }$ from **a** and *solid black square* are $Q_{s}$ values from 2011 and 2012 (they are identical values at this scale of view). *Star symbol* is $Q_{s}$ for 2012 extracted from 2013 $s_{i \cdot }$ data at the audit the five audit locations of 2012 and likewise for the pentagon symbol for 2011. *Grey bars* in **b** represent the sample space greater than $Q_{s}$ in 2011 and 2012

The univariate distribution of $s_{i \cdot }$ exhibits bimodality in 2013 (Fig. 5a). Spatially, this bimodality is evident in the map of $s_{i \cdot }$ (Fig. 4). In 2013, the observed $Q_{s}$ was 2.897. The observed $Q_{s}$ was 3.097 in 2011 and 3.094 in 2012 (Fig. 5b). Extracting $s_{i \cdot }$ from the 2013 dataset at the same block locations that were audited in 2011 and 2012, yields $Q_{s}$ values of 2.73 and 2.86 respectively (Fig. 5b).

Simulating 100,000 random draws of 5 without replacement from the 167 audit locations in 2013, the range of possible $Q_{s}$ is $2.00 \le s_{i \cdot } \le 3.66$ with ninety-five percent falling in the interval of $2.44 \le s_{i \cdot } \le 3.34$. The observed $Q_{s}$ in 2013 was 2.897 (±0.224). To assess the representativeness of the 2012 random sampling method, the observed $Q_{s}$ of 3.094 would occur at least 19.6% of the time when taking 5 random audit locations in that neighbourhood. Likewise, with draws of 4 samples, the 2011 $Q_{s}$ of 3.097 would be exceeded 22.1% of the time. In general, the observed $Q_{s}$ in 2011–2012 based on four or five random samples of $s_{i \cdot }$ are very likely to occur for this neighbourhood using the sampling methodology based on zoning density.

### Comparison with health determinants

In both years, $Q_{s}$ exhibits a positive significant correlation with both SES and, in 2012, with moderate or high physical activity (IPAQ) (Table 5). The correlation between $Q_{s}$ and self-reported overweight or obese (BMI) is significant and negative in both years (Table 5). In both 2011 and 2012, neighbourhoods ranking higher aesthetically are more likely to also possess high SES. Likewise, a highly aesthetic neighbourhood was associated with lower BMI and to a lesser extent a higher IPAQ.

**Table 5:** Spearman’s rank correlation coefficients, ρ, with bootstrapped 95% confidence intervals in brackets, between ${\mathbf{Q}}_{{\mathbf{s}}}$ in (2011, 2012) and SES, self-reported overweight or obese (BMI) and moderately or highly active (IPAQ)

	SES (n = 15)	BMI (n = 13)	IPAQ (n = 12)
$Q_{s}$ 2011	0.72 (*0*.*0012*) [0.2500, 0.9449]	−0.50 (*0*.*0425*) [−0.8571, 0.0467]	0.45 (*0*.*0752*) [−0.1739, 0.9091]
$Q_{s}$. 2012	0.60 (*0*.*0079*) [0.1320, 0.8448]	−0.65 (*0*.*0098*) [−0.9222, 0.0196]	0.69 (*0*.*0083*) [0.0571, 0.9423]

Empirical *p* values are in parentheses and were determined by 9999 permutations of ${{Q}}_{{{s}}}$

## Discussion

### Internal consistency

The aesthetics SSOI possesses acceptable to good internal consistency and the SSOI items within the neighbourhood aesthetics observational tool are sufficiently measuring and evaluating the same construct. Item-total correlation (ITC) values were acceptable to good in all years. The quality of pedestrian infrastructure had a weak ITC in all field seasons. The weak ITC may reflect the idea that pedestrian infrastructure is more indicative of physical disorder, rather than a direct indicator of aesthetics. The lower ITC values for cleanliness, the presence of trees and quality of trees in 2012 is most likely due to the timing of the field audit in that year. The observations in August of 2012 were during a prolonged drought with the driest July on record and the driest year on record in Ottawa. The condition of lawns and trees were affected by significant browning and/or leaf loss and this in-turn affected the perceptions of overall cleanliness and tree quality. These same items also exhibit the lowest and non-significant interrater reliability in 2012. The auditors had some difficulty in assessing these items based on their reference photos and VE training using non-drought conditions. Overall, however, the SSOI is capturing and evaluating the same construct(s).

### Interrater reliability

Overall, interrater reliability was greater for most SSOI items in 2011. We believe that this effect is due to the intervention methods applied in 2011. Three neighbourhoods were observed each week and prior to the next observation session, IRR was calculated to determine which SSOI items required improvement and subsequently followed up with mock training using Google Street View. The cumulative effect of these interventions was gradual improvement in IRR for SSOI items that showed improved consensus building. Thus, sequential interventions may be more effective than a single pre-audit period of training. Pedestrian infrastructure was problematic in 2013 where one auditor found the SSOI item not applicable far more often than the other.

### Neighbourhood average aesthetics index scores ($Q_{s}$) in 2011 and 2012

Except for two neighbourhoods, a total of five neighbourhoods with lowest SES also have the lowest neighbourhood average aesthetics index scores ($Q_{s}$). Moreover, three neighbourhoods which have high SES were ranked in the top five $Q_{s}$ values in both years. However, there were several exceptions that provide important information. For example, Billings Bridge, a neighbourhood in the bottom half of SES rankings, unexpectedly received high $Q_{s}$ values in both audits. That result suggests that SES may not always be indicative of a neighbourhood’s aesthetic appeal. In contrast, the neighbourhood of Borden Farm—Stewart Farm—Parkwood Hills—Fisher Glen, ranked much higher in 2012 despite having lower SES. The neighbourhood of Glen Cairn—Kanata South Business Park ranked highly in 2011 and much lower in 2012. In this case, examination of the field reference photos in the two years pointed out that the audit locations in 2012 were hard-hit by the 2012 drought in Ottawa, affecting landscaping and trees.

### Sampling approach

Despite having different auditors in all three years, $Q_{s}$ values within the neighbourhood with 167 block observations (Overbrook-McArthur) were very similar and show that the observed $Q_{s}$ values from the random sampling in 2011–2012 are not significantly different from the 2013 $Q_{s}$ across 167 full blocks. This suggests that taking 4 or 5 random audit locations within a neighbourhood stratified by residential zoning type can sufficiently characterize neighbourhood aesthetics.

We choose to observe all 167 blocks in the Overbrook-McArthur neighbourhood to validate our sampling regime because this neighbourhood is heterogeneous. Overbrook-McArthur’s spatial variability in $s_{i \cdot }$ (Fig. 4) is typical of a neighbourhood that has been undergoing gentrification. Over the past two decades, gentrification began with the western portion of the neighbourhood adjacent to the Rideau River and has more recently increased in the section east of the Vanier Parkway and west of St. Laurent Boulevard. These gentrified regions have the highest $s_{i \cdot }$. Lower $s_{i \cdot }$ values are apparent within the center of the neighbourhood, a region that contains row housing and low-income housing units (two middle panoramas in Fig. 4). The spatial variability of $s_{i \cdot }$ is reflected in the bimodality of the frequency distribution of $s_{i \cdot }$ (Fig. 5a) and this bimodality is caused by the pattern of gentrification.

The $Q_{s}$ ranking of CFB Rockcliffe-NRC in 2011 and 2012 was drastically different and requires explanation. Exceptionally in 2011, only three residential locations were audited. The fourth location was within a former medium-density residential military housing unit that was undergoing demolition. That fact was unknown when establishing the audit locations. In 2012, four of five audit locations were in the high density zoning areas, whereas in 2011 the audit locations occurred equally within high, medium and low density zoning. Moreover, much of this neighbourhood is largely green space that was reclaimed from a former Royal Canadian Air Force base.

### Comparison with health determinants

The neighbourhood average aesthetics index score ($Q_{s}$) relations with health determinants are generally consistent with expectations. However, given the small sample size of 15 neighbourhoods these observations largely provide impetus for further research and hypothesis testing with a larger number of neighbourhoods. Specifically, the efficient and effective method of auditing described herein can facilitate future research wishing to conduct spatially large scale studies in a temporally and financially responsible manner. In addition, with a larger sample, determination of MAUP induced zoning and spatial scale effects on $Q_{s}$ variation could be assessed [58].

Support from other research lends further strength to the comparison of aesthetics and health determinants (i.e., BMI and physical activity) and offers opportunities for future research. The connection between aesthetics and health is commonly observed in research on physical activity, such as walkability, which, at the ecologic level, can be directly correlated with health outcomes [13, 35, 59, 60]. Walkability is the extent to which the built environment supports and encourages walking and has been linked to physical health with benefits such as improved BMI and cardiovascular fitness [59]. Neighbourhood aesthetics were found to be a significant predictor of walkability and physical activity in a study spanning 11 countries [13]. For instance, people are more likely to walk and be otherwise physically active in aesthetically appealing neighbourhoods [19, 61, 62].

### Efficiency of the mobile GIS equipment

The mobile GIS technology proved to be an extremely valuable, efficient, and effective combination. Mobile technology provides a medium that enables auditors to efficiently travel to and observe each of the randomly selected audit locations, thereby streamlining data collection and data entry. As such, more time can be invested in subsequent data analysis.

The SSOI only had to be entered into one iPad and then shared as a GIS Kit feature class among everyone within the audit team. That process allowed for standardization of the SSOI across all devices. Moreover, because all data was digitally stored on the iPad in a GIS friendly format, data could be uploaded or e-mailed at the end of each audit session thereby minimizing risks of data loss or corruption if a device was damaged. The capture of geotagged photographs within each audit location and stored within the geographic feature table of each point were useful in understanding the effects of drought in 2012 on some SSOI items.

The iPad contains many other applications that were valuable to the current study and aided in the efficiency and effectiveness of data collection. One of the biggest advantages of using the iPad is its use of task-specific applications and software. The capability of GIS Kit to cache Google Maps and satellite imagery, along with its GPS navigator, provided an easy to use method of navigation which enabled the auditors to efficiently travel to audit locations without an internet connection. GPS navigation was particularly useful for the spatially random sampling design used in this study, since audit locations are scattered across urban space. The auditors could easily ask for directions to the next audit location and follow the computed route. Furthermore, during training sessions, the ability to modify items and SSOI descriptions in the field during an audit and then sharing the modified feature class was convenient in preparing the devices for use in the sampling.

There are a range of other options for georeferenced field data collection, and, while they cannot all be reviewed here, some common options are ArcPad for ArcGIS (http://www.esri.com/software/arcgis/arcpad); however, a licensing fee and ArcGIS desktop are required as well custom development in ArcPad. ArcGIS Collector and, in particular, Survey123 (https://survey123.arcgis.com/) by ESRI Inc. can work in either Android and iOS or a web browser but do require a paid institutional subscription to ArcGIS Online. Maptionairre (https://maptionnaire.com/) is a software as a service (SaaS) that can be purchased by project or on a continuous basis. Similar functionality can be achieved using open source software such as the web form based Kobo Toolbox (http://www.kobotoolbox.org/) or QField (http://www.qfield.org/) for Android that operates within the QGIS ecosystem but does have a larger learning curve.

### Generalization

There is an increased need for built environment audits that consider non-US contexts (where a large number of observational audits originate) [63]. Highlighting this need are the many cultural and social differences between the US and other parts of the world including both Canada and Europe. For instance, using Canada as an example, there is a significant difference in the levels of crime and minority segregation in neighbourhoods and both tend to be higher in the United States [5]. Furthermore, Canadian differences in the experience of low income households, for example, presents a challenge when applying U.S. based neighbourhood studies [64]. Our study is applicable to other Canadian contexts, particularly regarding the underlying mechanisms and implications (e.g., policy formulation), and the methods can equally be applied to other countries and regions in the Northern and Southern Hemisphere with similar populations, urban development, built environments, and land use features.

## Limitations

A common limitation of neighbourhood observational field audits is weather conditions [5]. To ensure high reliability and validity, it is best to observe neighbourhoods under the same environmental conditions; however, this can be difficult, especially at certain times of the year. The observations in 2011 took place during the month of November; a time when the weather is cooler, trees have less leaves, and snow can be common. In 2012, the field audit took place during the final stages of a major drought. Although steps were taken to prevent bias due to weather, it is recommended that future studies evaluate aesthetics in different seasons (e.g. spring or summer) to determine the variability of aesthetic features at different times of the year and their potential impact on perception and measurement. In a Canadian context, SSOIs that account for winter require considerable research. One advantage of VE audits, such as those using Google Street View, is that they can minimize some weather induced biases because almost all imagery is from the summer months in the Northern Hemisphere. It may be advantageous to utilize both virtual and field audits simultaneously to assess seasonal biases if temporal discrepancies in street view imagery can be controlled.

Although there are many benefits to utilizing the iPad in the current study, there were also minor limitations with the GIS Kit application. For instance, formatting long SSOI item titles to fit the GIS Kit data entry column was difficult (Fig. 1). The SSOI item called “cleanliness” was actually “cleanliness of streets and properties”, but placing long titles on the data entry column in GIS Kit made data entry cumbersome. Another limitation of GIS Kit involves the clarity and relevancy of the Google Street or Satellite caches (maps). Although rare, reliance on 3rd party mapping systems, like Google Maps, meant that some audit locations were within sections of a neighbourhood that contained new developments that were not yet integrated into Google Maps. Thus, on occasion, the ability of the auditor to efficiently travel to the predetermined area was affected. Another minor concern, while not unique to the iPad, is the touch screen. This technology is extremely sensitive; therefore, auditors must be very aware of when they are touching the screen because they could unintentionally alter the collected data with an unintended touch of a finger or knuckle. A final concern, more so than a limitation, is the learning curve required to use an iPad and the GIS Kit application. The iPad, and more so GIS Kit, requires some familiarity and training to be able to use both efficiently and effectively. Although there are some limitations to utilizing the iPad and GIS Kit in the current study, the benefits and potential of this technology far outweigh the limitations. Future studies are recommended and encouraged to utilize mobile GIS technologies and applications to improve the efficiency of research design execution.

## Conclusion

With the expansion and growth of research on neighbourhood characteristics in recent years there is an increased need for direct observational field audits, and specifically, research that focuses on the aesthetic features of neighbourhoods [2–4, 19]. This focus will also provide a more complete and contextual perspective for neighbourhood research. The current study addressed the need for direct observational research by showing that a simple SSOI together with minimal sampling and mobile GIS technology can be effective for rapid BE audits and evaluation. The need for direct observational research is not only relevant to Canadian settings [5] but is applicable to other countries and regions in the Northern and Southern Hemisphere with similar populations, urban development, built environments, and land use features.

The current study evaluates a new and effective collection method that is relevant to several disciplines and presents many potential research opportunities. A manifestation of this interdisciplinary relationship has already occurred. For example, the City of Ottawa Crime Stoppers program expressed interest in utilizing the new direct observation method and SSOI to examine neighbourhood aesthetic levels in relation to crime rates in Ottawa neighbourhoods. Based on the outcome, efforts to restore or improve neighbourhood aesthetics could be implemented and these policies may create more enjoyable neighbourhoods for residents, which in turn would actively promote the mental and physical well-being of residents.

## Abbreviations

GIS: geographic information systems
SSOI: systematic social observation instrument
SES: socioeconomic status
BMI: body mass index
GPS: global positioning system
KML files: Keyhole Markup Language, an open source ASCII format for geographic data
R1 to R5: types of residential dwellings
IRR: interrater reliability
ICC: intraclass correlation coefficient
ITC: item-total correlation
IPAQ: International Physical Activity Questionnaire
CFB Rockcliffe NRC: Canadian Forces Base Rockcliffe, National Research Council
MAUP: modifiable areal unit problem
ArcPad: ESRI Inc. mobile windows CE, windows phone application for mobile geographic data collection
iOS: an operating system for mobile devices used by Apple Inc.
